# Rectal Tonsil in a Patient With Positive Syphilis Serology

**DOI:** 10.7759/cureus.23812

**Published:** 2022-04-04

**Authors:** Daniel D Cain, Kathleen Martin, Bradley Gibson

**Affiliations:** 1 Medical School, University of Pikeville, Kentucky College of Osteopathic Medicine, Pikeville, USA; 2 Gastroenterology, Saint Joseph Medical Center, Lexington, USA; 3 Hematopathology, Pathology and Cytology Labs Inc., Lexington, USA

**Keywords:** histology, gastroenterology, syphilis, lymphoid polyp, rectal tonsil

## Abstract

Rectal tonsils are an abnormal reactive proliferation of lymphoid tissue in the rectum. Typical lymphoid tissue of the colon and rectum can proliferate with an increased number of germinal centers in response to exposure to an antigen in the GI tract. This response, in rare cases, escalates to the proliferation of a lymphoid mass known as a rectal tonsil. Here, we present a case of a 46-year-old woman with iron deficiency anemia who underwent colonoscopy with incidental finding of a rectal tonsil. This report discusses initial clinical workup, colonoscopy findings, pathological analysis, subsequent testing, and surgical removal of the identified mass and this rare finding.

## Introduction

Rectal tonsils present when there is abnormally increased growth of reactive lymphoid tissue in the rectum resulting in the formation of a mass. The etiology of this occurrence is unknown but has been found to occur in patients with other underlying disorders like diverticulosis [1-3]. The cause of this reactive growth is thought to be secondary to an antigen that has been encountered by the local lymphoid tissue although it has not been found to result from a specific antigen [4]. Histologically, these masses can be differentiated from a malignant etiology by the presence of structurally normal lymphoid tissue [1]. Rectal tonsils have been reported in other cases where underlying infections such as Epstein-Barr virus (EBV) or chlamydia were identified, but have not been reported in a patient who is *Treponema pallidum* antibody-positive until now [1,4,5].

## Case presentation

**Figure 1:**
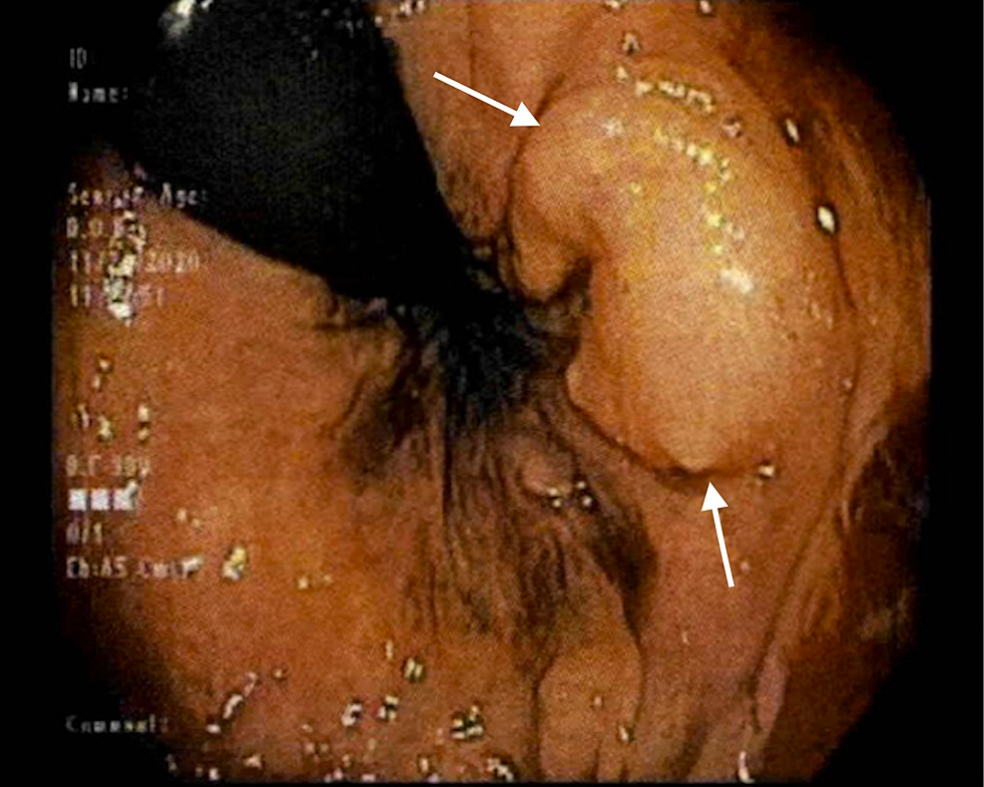
A 1cm submucosal lesion noted on the left side of the rectum.

A 46-year-old African American woman with history significant for iron deficiency anemia was referred for colonoscopy to evaluate for a potential source of bleeding. Prior to scope insertion, rectal exam identified a 1cm submucosal mass just inside the anal verge on the left side proximal to the anorectal ring (Figure 1). The lesion was unroofed via hot snare, and a sample was obtained with cold snare. The mass could not be fully visualized or removed due to its submucosal nature, though carcinoid versus gastrointestinal stromal tumor (GIST) was suspected initially based on the whitish appearance of the submucosal tissue and firm nature on palpation.

The patient was referred to a colorectal surgeon for complete excision of the mass. Histopathology of the initial sample (Sample 1) collected at colonoscopy showed an atypical lymphoid population, described below. Serological testing was positive for *T. pallidum* antibody test and *T. pallidum* antibody enzyme immunoassay (EIA), though rapid plasma reagin (RPR) testing was negative. Upon further questioning, the patient denied any signs or symptoms of a previous sexually transmitted infection (STI). Three weeks after colonoscopy, the colorectal surgeon removed the submucosal mass via transanal excision and sent the specimen (Sample 2) to pathology. The patient was also treated with a course of bicillin 2.4 million units administered intramuscularly at three separate visits at roughly one-week intervals. This was done due to her reactive treponemal tests, and her denying having had previous treatment.

Histopathology

Sample 1 consisted of two portions of tan-brown soft tissue obtained during colonoscopy. It was significant for atypical lymphoid proliferation demonstrating extensive submucosal proliferation of predominantly mature small lymphocytes that infiltrated and caused expansion of the lamina propria. No lymphoepithelial lesions were seen. Immunohistochemistry showed expanded B-cell follicles with associated follicular dendritic cell networks, expanded marginal zones, and no aberrant immunophenotypic expression. B-cell gene rearrangement studies revealed no evidence of clonality.

**Figure 2:**
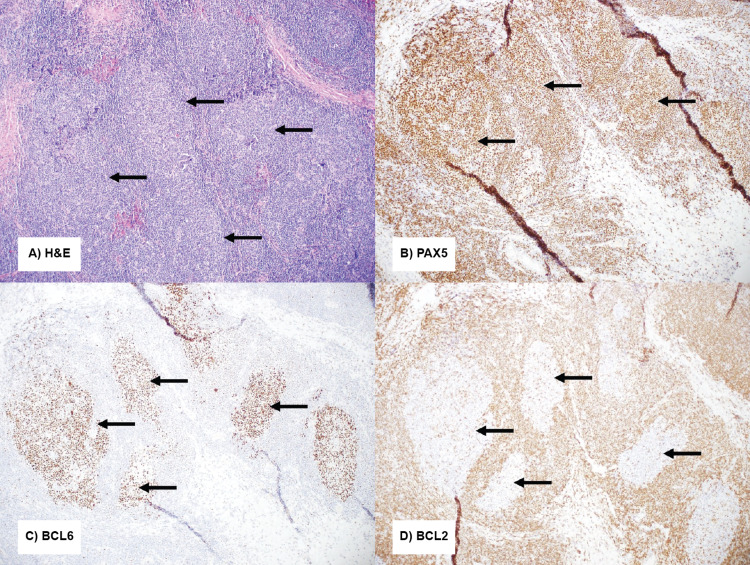
(A) H&E-stained sections of the resection specimen showed expanded secondary follicles (see arrows); (B) PAX5 showed B-cell follicles (see arrows); (C) BCL6 highlighted germinal centers (see arrows); (D) BCL2 was negative in germinal center B-cells (see arrows). H&E: hematoxylin and eosin stain; BCL6: B-cell lymphoma 6; BCL2: B-cell lymphoma 2

Sample 2 obtained during transanal excision of the rectal mass provided a greater volume of the lymphoid population for evaluation, which showed organized and expanded lymphoid follicles with loose fibrous banding. Flow cytometry showed polyclonal B-cells and unremarkable T-cells. Immunohistochemistry demonstrated findings in keeping with reactive lymphoid tissue, including well-demarcated BCL6+/BCL2- germinal centers associated with CD23+ follicular dendritic cell networks, surrounded by mildly expanded but polarized marginal zones (Figure 2).

## Discussion

The report done by Hong et al. demonstrated that rectal tonsils were found in patients of all ages, races, and sexes and that the morphology and presentation can vary widely [1]. The findings of this case lead us to hypothesize that the patient’s syphilis may have played a role in the reactive lymphoid tissue found within the submucosal mass. This infection could cause the observed lymphoid response as syphilis causes increased germinal center proliferation and lymphoid response [1-3,6]. Similar cases have been described in patients with other underlying infections including EBV, *Chlamydia trachomatis*, and tuberculosis [1,4,5]. A possible correlation between increased lymphoid tissue of the digestive tract and diverticulosis has also been described [3]. Rectal tonsils have been documented to mimic lymphoma. However, the normal architecture of the lymphoid tissue and lack of aberrant immunophenotypic expression by immunohistochemistry and flow cytometry ruled out the possibility of lymphoma [7]. Further serologic workup could have been done, but it was determined that prompt treatment of the patient was in her best interest. The patient was treated with three doses of bicillin and continued follow-up with the primary care physician (PCP). 

## Conclusions

In conclusion, the diagnosis of rectal tonsils should be considered in the differential diagnosis when a submucosal mass is discovered in the colon or rectum. The additional testing needed to rule out malignancy should include wide margin excision as well as immunohistology and flow cytometry. This interesting case of a rectal tonsil being diagnosed in a patient with positive syphilis serology with no prior treatment is a unique addition to the literature.
